# Machine learning models to predict micronutrient profile in food after processing

**DOI:** 10.1016/j.crfs.2023.100500

**Published:** 2023-04-14

**Authors:** Tarini Naravane, Ilias Tagkopoulos

**Affiliations:** a Department of Computer Science, University of California at Davis, United States; b Biological Systems Engineering, University of California at Davis, United States; c Genome Center, University of California at Davis, United States

**Keywords:** Food processing, Nutritional profile, Machine learning, Prediction models, Data science, Food composition dataset

## Abstract

The information on nutritional profile of cooked foods is important to both food manufacturers and consumers, and a major challenge to obtaining precise information is the inherent variation in composition across biological samples of any given raw ingredient. The ideal solution would address precision and generability, but the current solutions are limited in their capabilities; analytical methods are too costly to scale, retention-factor based methods are scalable but approximate, and kinetic models are bespoke to a food and nutrient. We provide an alternate solution that predicts the micronutrient profile in cooked food from the raw food composition, and for multiple foods. The prediction model is trained on an existing food composition dataset and has a 31% lower error on average (across all foods, processes and nutrients) than predictions obtained using the baseline method of retention-factors. Our results argue that data scaling and transformation prior to training the models is important to mitigate any yield bias. This study demonstrates the potential of machine learning methods over current solutions, and additionally provides guidance for the future generation of food composition data, specifically for sampling approach, data quality checks, and data representation standards.

## Highlights

• ML models can predict the nutrients in cooked food with less error than baselines.
• The models can identify subcategories of the data that have the best results. The leafy greens category is the most predictable of the plant-based foods in the dataset.
• Beef cuts are most predictable of the animal-based foods in the dataset.
• The cooked food composition data is biased due to missing yield factors and was addressed by data scaling methods.
• Data science methods can be applied to guide future data generation efforts.

## Graphical abstract

**Figure:**
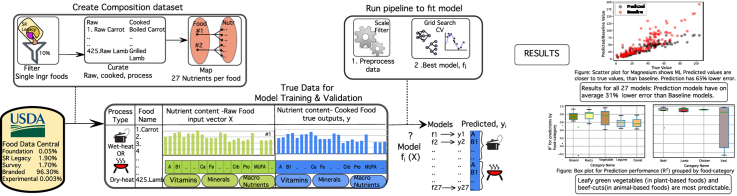

## 1 Introduction

Food processing, such as fermentation, baking, or even boiling alters the chemical composition of food, often in unpredictable ways from the raw to the finished state. This is due to the unresolved chemical and structural complexity of the food and the physio-chemical transformation mechanisms that occur during processing (Capuano et al., 2018). Yet in spite of these challenges, the objectives for prediction models are compelling which include sensory properties (Parker, 2013) such as aroma, texture, taste, etc., and nutrient profiles, and here we address the latter. The models in use currently, simplify the inherent complexity and instead predict the content of a nutrient based on only a few parameters. For instance, kinetic modelling based on experimental data for any given food establishes the relationship between nutrient concentration, time and temperature conditions (van Boekel, 2008; Martinus, 2022; Bajaj and Singhal, 2020; Peleg et al., 2018). This can then be applied to compute concentrations, for example predicting vitamin C (ascorbic acid) content in processed orange juice (Peleg et al., 2018). Another approach to compute post-process nutrition composition, is to apply retention factors (RF) which are based on analytical composition data on a representative set of foods and processes. RF-based computation is used widely by food manufacturers for nutrition labels, and by USDA's dietary survey group to calculate nutrient intakes that investigators may use to determine correlations between intake and health outcomes (Nutrient retention factors, 2022). However, all of these methods have limited potential. Kinetic models are difficult to scale up to capturing more food and processing parameters, as these measurements are time-consuming, expensive (Ling et al., 2015) and have many experimental challenges such as certain chemicals which degrade rapidly. RF-based methods in practice inevitably under or overestimate the nutrient content in a particular instance, since any single RF is representative of several foods and a cooking method. Here we address the challenge that our knowledge of composition and reactions of food systems is limited, which inevitably manifests to such incomplete or underdetermined models. This can be at least partially addressed with predictive machine learning (ML) methods that can learn the multi-parametric transformation patterns between the compositions of raw and cooked foods, from experimental data across diverse foods and cooking methods.

The application of ML to food science data is at an early stage, yet it has been successful in generalizing across a variety of prediction tasks when trained on relevant datasets. We now give a brief summary of some recent work on prediction of nutrient profiles or properties of food and then explain how it informs our work. Classifier models have been applied to predict sensory properties from the molecular structure, such as bitter (Charoenkwan et al., 2021; Dagan-Wiener et al., 2017) and sweet (Zhong et al., 2013; Tuwani et al., 2019) and aroma labels (Sanchez-Lengeling et al., 2019). A number of food quality classifiers use hyperspectral data, for example for the freshness classification of shrimp (Yu et al., 2018), detection of adulteration in red meat products (Al-Sarayreh et al., 2018) or detection of damaged/bruised fruits and vegetables (Wang et al., 2018; Liu et al., 2018). Several models have addressed attributes related to nutrient profiles. The P_NUT model uses natural language processing (NLP) methods and predicts the macronutrient (proteins, fats and carbohydrates) content of foods from a text description of the food (Ispirova et al., 2020) and a more recent version can predict macronutrients, from a recipe (Ispirova et al., 2021). USDA investigators predicted the content of three label nutrients (carbohydrates, protein and sodium) in processed foods from the ingredient list (Ma et al., 2021), using the Branded Foods datatype in Food Data Central (FDC) (National Agricultural Library, 2019). Several projects predicted nutrient contents from the composition data; nutrient content was predicted for the missing values in food composition data (Gjorshoska et al., 2022), lactose content was predicted in dietary recall database (Chin et al., 2019), fiber content was predicted for commercially processed foods (Davies et al., 2021). In the context of food processing a food was assigned a label of the degree of processing based on the composition data (Menichetti et al., 2021), and the foods were either raw or industrially processed and the four possible labels were as per the NOVA (Moubarac et al., 2014)system ranging from minimally-processed to ultra-processed. Availability of datasets with high quality data for training and testing is essential, and databases such as BitterDB (Dagan-Wiener et al., 2019; Wiener et al., 2012), FlavorDB (Garg et al., 2018), FooDB (FooDB., 2022), SuperSweet (Ahmed et al., 2011), Fenaroli (Burdock, 2016), GoodScents (The Good Scents Company Information System, 2022), FDC (National Agricultural Library, 2019) as well as specifically curated datasets of hyperspectral images may contribute to this end.

**Fig. 1:**
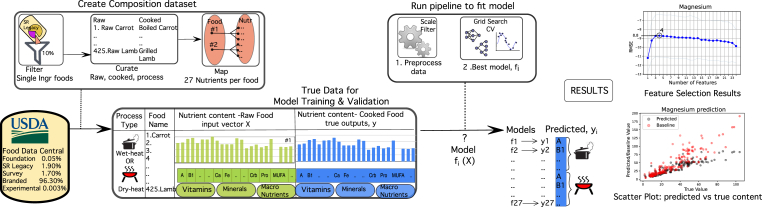
**Overview of architecture (left to right) from data selection to prediction results.** Single ingredient foods are selected from SR legacy (one of the five data types in FDC), and then organized by pair (raw,cooked) and cooking process type. Cooking processes include boiling and steaming which are grouped into wet heat processes (WH) and broiling, grilling, and roasting which are grouped into dry heat processes (DH). Foods are mapped to composition, with 27 components per food. Models are trained from composition data, such that the input feature is the composition of the raw food, and each model is trained separately for every micronutrient in the cooked food. Models are trained separately for both process types, with 14 for WH and 13 for DH (excluding vitamin C predictor model). Prior to model fitting, the composition data is scaled and filtered. Model fitting uses a grid search cross validation approach, such that there are 12,336 regressor models. The best model has the least error, RMSE. Then predicted composition is compared to the actual (ground truth) composition in two results. The feature selection result is the performance (RMSE) analysis against the feature (input features) size. The scatter plot for prediction of magnesium content shows the both the prediction (black dots) and baseline (red dots) values on the Y axis, versus the actual values (X axis). (For interpretation of the references to colour in this figure legend, the reader is referred to the Web version of this article.)

This body of prior research implies that there is a complex interdependence between the chemical components of the food and supports the hypothesis of our work, that the transformation patterns in food composition due to a variety of processes can be learnt. Here, we have constructed ML models that predict food micronutrient (specifically seven vitamins and seven minerals) composition after processing (Fig. 1). We have curated a sample of 820 single-ingredient foods in the raw and cooked states, for five basic cooking processes namely steaming, boiling, grilling, broiling, and roasting from FDC. (Our aim is to model basic single-step cooking processes, and we did not consider multi-step processes as in recipes or industrial processes.) We then trained regressors per nutrient and per process that have achieved a correlation (R^2^) between actual and predicted micronutrient values that range from 0.42 to 0.95 (outliers are −0.42, −0.09,0.13 and 0.23).

## 2 Materials and methods

### 2.1 Dataset

**Fig. 2:**
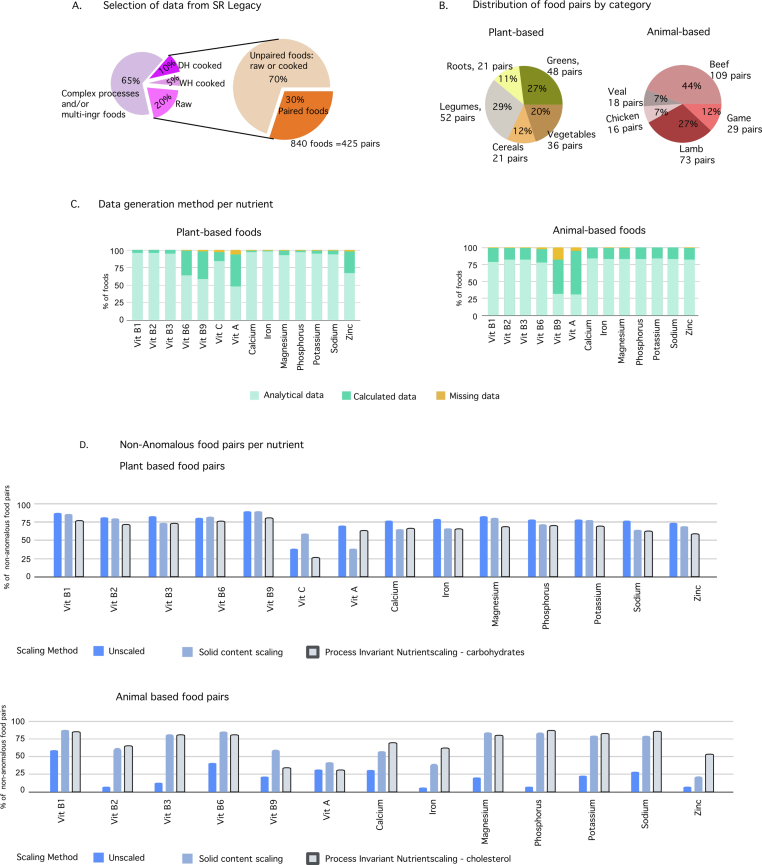
**Data Review.** (A) Out of 7793 foods in the SR Legacy datatype in FDC dataset, 2724 (35%) are single ingredient foods. Within that set, we identified 425 pairs of raw-cooked single ingredient foods. (B) The food pairs per category for plant-based and animal-based foods. There are a total of 178 pairs of plant-based foods and 247 pairs of animal-based foods. (C) The food-pair distribution by the method of data generation. (D) Comparing the percentage of food-pairs of non-anomalous data by scaling method.

We downloaded the composition dataset of 7793 foods from the Standard Reference (SR) legacy dataset (USDA National Nutrient Database for Standard Reference, 2022), which is the most suitable of the five data sets in FDC (Fig. 1; as of November 2021), since it is aligned with our objectives. The SR dataset has composition data for both raw and cooked food samples for single ingredients and is intended for application in public health initiatives such as the assessment of nutrient intakes for the purpose of national nutrition monitoring, in creating meal plans in schools and day-care centers, in product development and labeling by manufacturers. The composition data for the foods in SR is obtained from three sources; analytical experiments, analytical data from literature, and calculations based on the analytical data for example composition data on butterhead lettuce is calculated from composition of leafy green lettuce which is a “similar food” (Haytowitz and Pehrsson, 2020; Haytowitz et al., 2009). The complete list of composition source types is in Supplementary materials. The four other data sets with composition data in FDC are; Foundation foods with single-ingredients foods and mostly only raw foods and the aim is to provide high quality data on raw ingredients with relevant meta-data as a precedent for future data sets, Experimental foods with the aim of studying certain production methods (such as environmental growing conditions) for their effects on composition, FNDDS where the composition data is calculated such that it is representative of the diets reported in the What We Eat in America survey (and not analytically measured, for example “asparagus cooked with fat” is a sum of the composition of cooked asparagus and composition of a non-specific fat which is a weighted sum of various consumed fats) and Branded Foods datasets has commercially available industrially processed foods (Fukagawa et al., 2022). Fig. 2 gives a breakdown of the SR dataset and our selection, where there are 1546 raw foods, 384 cooked foods by the wet heat process, 806 cooked foods by the dry heat process and the remaining 5057 foods were made by other processes. For our models, we selected a subset of the SR dataset according to the following criteria. We matched raw/cooked food pairs, where the raw foods were a single ingredient harvested from a plant or from an animal (includes butchery products), and the cooked food was the outcome of the raw food treated to wet (boiling, steaming), or dry (roasting, grilling, broiling) heat processes. Foods were excluded from the dataset if either there was no single-ingredient raw food corresponding to the cooked food and vice-versa, or the foods had several ingredients and produced by a multi-step process like ‘Luncheon meat, pork and chicken, minced, canned, includes SPAM Lite’, ‘Bread, banana, prepared from recipe, made with margarine’. We excluded processes which have added ingredients such as oil for frying and stir-frying although they these are common methods for cooking since we did not have data on the composition of the oil used in the process. We included boiling and steaming (simple aqueous, i.e., wet heat processes), as well as roasting, broiling and grilling (dry heat processes). This resulted in 840 foods total in the dataset, with 178 and 247 pairs from wet and dry heat processes, respectively. In this dataset, all plant-based foods were cooked by wet heat process (WH), and all animal-based foods by a dry heat (DH) process. (This congruence is a limitation in this dataset and is addressed in the Discussion.) The categorical breakdown of the number of pairs for plant-based and animal-based foods is shown in Fig. 2.

The composition data consists of content values for up to 232 ‘chemical constituents’ or ‘components’, which include specific chemicals (vitamins, amino-acids, fatty acids, etc.) and aggregated chemicals or chemical groups (total fats, total proteins, etc.) for every food. Here, we selected the components that are reported for at least 80% of the foods in our dataset. This resulted in 27 components per food, namely nine vitamins, 10 minerals, water, and seven aggregates of total protein, total carbohydrates and various fat categories (Supplementary materials). This composition data was used to train the prediction models where the input feature set to every model is the content of the 27 components in the raw food and the outputs are the contents of the 14 micronutrients in the cooked food. For this study, the macronutrient composition data in the cooked food is not predicted by the model, however this data is important for the data preprocessing explained next. Prior to model fitting, the composition data should be preprocessed to adjust for the bias resulting from the conventional format of representing nutrient contents per 100 g of a food sample. In actuality, the cooked food sample would have a higher yield in the wet-heat process compared to the raw food sample primarily due to the gain of water and a lower yield in the dry-heat process due to the loss of fat and water. Scaling the true weight to the 100 g representation in case of the higher yield creates an underrepresentation of the solid components. In the case of the lower yield the 100 g representation creates an overrepresentation, which was observed as higher nutrient contents in the cooked food sample relative to the raw food sample. Ideally the data preprocessing would reverse this scaling effect. We use two different scaling methods, solid content scaling in Equations (1), (2)) and process-invariant nutrient scaling in Equations (3), (4)). For the solid content scaling (SCS), the assumption is made that the water and fat contents remain unchanged from the initial (raw) to final (cooked) state of the food, and as per Equation (1) the content in the raw food is set to match that in the cooked food. Then the contents of the other components in the raw food are scaled to compensate for the difference (R [water]-C [water]) while preserving their initial proportions as per Equation (2). Equations (1) and (2) are applied twice, once to equalize water and then to equalize fat, and the resulting scaled data is not affected by the order. This scaling method mitigates the over/under representation effect caused by gain/loss of water/fat. The second method attempts to identify the unknown yield factor (for the cooked food) as per Equation (3) and is based on identifying a nutrient that is largely invariant to processing. This factor is then used as per Equation (4) to derive the composition for the “true” weight of the cooked food corresponding to a 100-g sample of raw food. The concept of such a nutrient is an exception since processing creates the conditions for nutrient transformation through chemical reactions and loss through solubilizing and leaching in the water and fat. An exception is cholesterol in meat which is theoretically invariant to processing since it is in the muscle-cell membranes that are resistant to cooking loss. However, the experiments report a small loss (USDA National Nutrient Database for Standard Reference, 2022), so we also scale the data for a 5% loss and consider whether models are significantly different in reporting our results. The data for the cuts of beef used in this study is from experimental studies published by USDA where it is reported that contents of Iron and zinc contents were not significantly different in the raw and cooked beef (Roseland et al., 2018). There is no information on the components in plant-based foods. For confirmation of these hypotheses, all components are used in the PINS method and prediction performance is compared for both animal and plant-based foods. To be clear, the aim of these scaling methods is creation of alternate versions of the composition data that represent the yield information that was missing in the original FDC data. In the Results section we compare the model performance for these different versions of the data. A detailed explanation of scaling with examples is in the Supplementary Materials B.

In the Equations for scaling methods, *R* represents the raw food and *C* represents the cooked food, *R′* and *C’* represent the scaled data, and *X* is the generalized term for the components. In Equation (2), the summation term does not include water, and for the next step of equalizing the fat, the summation term would exclude water and fat.

(1) $$R^{\prime }\left[water\right]=C\left[water\right]$$

(2) $$R^{\prime }\left[X\right]=R\left[X\right]*\left(1+\frac{R\left[water\right]-C\left[water\right]}{\sum R\left[X\right]}\right)$$

(3) $$ScalingFactor=\frac{R\left[Cholesterol\right]}{C\left[Cholesterol\right]}$$

(4) $$C^{\prime }\left[X\right]=ScalingFactor*\;C\left[X\right]$$

All versions of the composition dataset include 425 pairs of foods, with 27 components, five processes (boiling, steaming, roasting, grilling and broiling), in two states (raw and cooked). (Supplementary materials).

### 2.2 Models

We trained models to predict the content of 14 micronutrients for which we had baseline retention factors in the cooked food. Of those, seven are vitamins, namely vitamin B1 (thiamin), vitamin B2 (riboflavin), vitamin B3 (niacin), vitamin B6 (pyridoxine), vitamin B9 (folate), vitamin C (ascorbic acid), vitamin A, and the other seven are minerals, namely calcium, iron, potassium, phosphorus, magnesium, sodium and zinc. We created separate models based on the process category (wet, dry), as these are fundamentally different processes, but not based on the actual process (e.g., boiling vs. steaming), as there are not sufficient data per process to avoid overfitting. All models have the same input, which is the composition of the raw food, as illustrated in Fig. 1. Other details that might be informative to the task (cooking time, temperature, water content) were not available in the SR legacy dataset, and consequently were not present in our dataset, or our model. Since vitamin C is not present in meats (which are all the foods for DH models), the dry heat models are only 13, for the other micronutrients, resulting in 27 models total (13 for DH and 14 for DH). These sets of WH and DH models were trained and tested on scaled variants of the dataset explained earlier. We applied a filtering step to the scaled datasets to select the pairs of foods where the nutrient being predicted was more in the raw food than in the cooked food. The unscaled data for the dry heat models and wet heat models was not filtered for this condition. So, each of the nutrient models were trained on different subsets of the data and is the reason that we did not have a single model to predict all nutrients. The effect of the data scaling and filtering on the predictive models is explained in the **Results**.

The best performing model (for any dataset variant) was selected based on a cross validation grid search across six regressor types (MLP, LASSO, Elastic Net, Gradient Boost, Random Forest, Decision Trees), each with a variety of hyperparameters totaling 12,336 regressors where the metric for the best model was the least root mean squared error (RMSE). This was done for each of the 27 models using the sklearn library (Pedregosa et al., 2011) and the best hyperparameters for each of the regressor types along with the RMSE is in Supplementary materials. We then performed a feature selection technique, a recursive feature elimination variant as described in the sequential feature selector function of the mlxtend package (Raschka, 2018). The model performances for data variants for the WH and DH process are compared in Table 1.

**Table 1:** **Comparing models trained on different data variants.** The prediction performance results for the models trained on data variants specified in the Methods are shown in this table. The metric for model performances is RMSE – Root mean squared error. A complete coverage of all performance for all PINS data is in Supplementary materials. Data Variants: Unscaled is the original data. SCS – Solid content scaling. PINS – Process Invariant Nutrient scaling and the specific nutrients is in parenthesis.

OUTPUT	WETHEAT		DRYHEAT				
	Unscaled	SCS	Unscaled	SCS	PINS (Zinc)	PINS(Iron)	PINS(Cholesterol)
Thiamine	0.04	0.02	0.03	0.02	0.01	0.01	0.01
Riboflavin	0.05	0.04	0.05	0.02	0.02	0.02	0.02
Niacin	0.48	0.21	0.87	0.68	0.46	0.60	0.45
B6	0.05	0.03	0.09	0.07	0.07	0.05	0.06
Folate	22.37	16.64	4.38	4.34	6.46	3.74	1.72
VitC	13.28	7.49	NA				
VitA	83.21	11.57	3.37	2.66	1.25	2.81	1.5
Calcium	22.17	14.28	4.33	3.5	2.41	1.36	1.81
Iron	0.6	0.30	0.33	0.31	0.14	0.00	0.16
Magnesium	11.19	6.66	5.05	3.98	2.19	2.22	2.33
Phosphorus	24.1	12.94	24.16	22.12	15.60	15.67	21.41
Potassium	101.9	46.95	48.87	39.2	27.49	30.95	32.23
Sodium	17.4	15.84	13.26	9.90	6.80	7.35	9.36
Zinc	0.2	0.10	0.67	0.51	0.00	0.38	0.29
AVERAGE	21.22	9.5	8.11	6.71	5.24	5.43	5.49

We assessed the predictive performance (RMSE) in comparison with two baseline models. The first is to naively assume that the dependent variable (the micronutrient to predict after cooking) is equal to its value in the raw food. This baseline serves as a comparison to a naïve regressor where the retention factor (RF) is 100%, i.e., the amount of the micronutrient after the heat process is the same as in the raw food. The second baseline was based on the USDA Retention Factors table, a common, standard model for the retention of nutrients after a process (Ling et al., 2015). The nutrient outputs were computed as a product of the RF for the specific nutrient and the content of that nutrient in the raw food. We use RSME, the coefficient of determination (R^2^), Pearson Correlation Coefficient (PCC), and Spearman Rank Correlation Coefficient (SRC) to assess the performance of our regressor model (Table 2 and Supplementary materials). At each case, we performed 5-fold cross validation runs, bootstrapped 50 times to avoid overfitting and increase the generalization potential of our classifiers. For a subset of foods (Supplementary materials), we provide a higher resolution baseline using retention factors from experimental studies in literature. Finally, we analyze the prediction performance through a breakdown of R^2^ by food category for plant-based foods (Leafy greens, Roots, Vegetables, Legumes, Cereals) and animal-based foods (Beef, Lamb, Chicken, Veal) as shown in Table 3. We do this by tagging every predicted micronutrient value by the category (associated with the food) and calculate the R^2^ for every group. This is repeated for all predictions, and the average R^2^ of a category is used to determine the best and worst performances in the plant-based and animal-based foods.

**Table 2:** **Results of prediction models compared to baselines.** The prediction scores (RMSE and R^2^) are the average of 50 runs, due to the inherent randomness in the models. [A] (RMSE) of best prediction models, compared to baseline (USDA's RF guide Version 6) model and naïve model (output content = input content). The most accurate prediction when compared to the True Data, among the the three prediction models (our model, Baseline and RF100) is highlighted with bold. The rel% column is calculated as: (baseline-predicted)/baseline × 100 1 B. Additional baseline model for vitamin C (ascorbic acid) and vitamin B9 (folate) using RF values from experiments on selected foods. 1C. The metric R^2^ (coefficient of determination) is scale invariant (as opposed to the RMSE) for ease in comparison across all predictions. The corresponding box plot is in Fig. 3.

Outputs	Wet heat (Steaming, Boiling)						Dry Heat (Broiling, Grilling, Roasting)			
	Avg + -Stdev		RMSE			Avg + -Stdev		RMSE		
	True Data	Predicted	Baseline	RF100	Rel %	True Data	Predicted	Baseline	RF100	Rel%
B1(Thiamine)	0.11+- 0.08	**0.02**	0.070	00.06	52.69	0.07+-0.04	**0.01**	0.02	0.04	14.69
B2 (Riboflavin)	0.08+-0.07	**0.04**	0.060	0.05	34.92	0.22+-0.1	**0.02**	0.03	0.04	28.03
B3(Niacin)	0.73+-0.54	**0.21**	0.550	0.89	54.38	4.52 +-1.2	**0.45**	0.53	1.19	15.04
B6	0.12+-0.11	**0.03**	0.060	0.09	25.84	0.29+-0.14	**0.06**	0.11	0.12	48.81
B9(Folate)	49.42+-48.81	**16.64**	51.480	26.35	60.12	10.99+-4.77	**1.72**	2.16	5.00	20.35
C	18.06+-21.96	**7.49**	7.540	15.09	−9.85		Not a significant source			
A	76.39+-118.23	11.57	35.750	**11.37**	33.28	3.83+-4.62	**1.50**	1.58	2.99	4.97
Calcium	43.68+-53.85	**14.28**	24.140	25.47	−6.71	9.47+-4.57	**1.81**	3.13	3.61	42.17
Iron	1.25+-0.97	**0.30**	0.940	0.54	52.29	1.74+-0.72	**0.16**	0.24	0.26	31.85
Magnesium	34.71+-22.1	**6.66**	25.630	9.18	65.56	17.96+-2.95	**2.33**	3.33	6.05	30.03
Phosphorus	75.89+-53.42	**12.94**	58.910	29.36	62.33	159.44+-24.64	21.41	**17.59**	34.89	−21.71
Potassium	268.57+-164.47	**46.95**	128.310	81.75	43.78	325.13+-86.05	32.23	**30.14**	66.83	−6.97
Sodium	15.63+-28.14	**15.84**	42.860	32.20	68.64	51.84+-11.34	9.36	**8.03**	16.64	−16.66
Zinc	0.62+-0.47	**0.10**	0.530	0.52	62.13	3.78+-1.67	**0.29**	0.53	0.52	45.38

Nutrient	Prediction model	Baseline (USDA RF table)	Baseline (RF from experiments)
Vitamin C	10.50	11.25	13.31
Folate	25.84	40.65	97.22

Outputs Metric:R2	Wet heat (Steaming, Boiling)			Dry Heat (Broiling, Grilling, Roasting)		
	Predicted	Baseline	RF100	Predicted	Baseline	RF100
B1(Thiamine)	0.80	0.26	−2.60	0.50	0.53	0.22
B2 (Riboflavin)	0.65	0.52	0.01	0.77	0.80	0.80
B3(Niacin)	0.73	−0.04	−1.30	0.80	0.80	0.91
B6	0.65	0.76	0.37	0.58	0.38	0.66
B9(Folate)	0.77	−0.12	−9.51	0.42	0.80	−1.09
VitC	0.76	0.83	0.29	Not a significant source		
VitA	0.95	0.91	0.87	0.59	0.86	0.98
Calcium	0.75	0.80	0.66	0.73	0.53	0.73
Iron	0.70	0.05	−1.02	0.76	0.80	0.30
Magnesium	0.82	−0.35	−2.04	0.13	−0.39	0.07
Phosphorus	0.78	−0.22	−1.04	−0.42	0.35	0.64
Potassium	0.71	0.39	−1.19	0.23	0.44	0.75
Sodium	0.64	−1.34	−11.47	−0.09	0.44	0.49
Zinc	0.79	−0.25	−1.03	0.89	0.90	0.97

**Table 3:** Various metrics (R^2^,RMSE,PCC) by category for plant-based foods Cereals do not have data for vitamin A and C predictions. Abbreviations are used for the predicted nutrient, Ca:Calcium, Fe:Iron, Mg:Magnesium, Ph:Phosphorus, K:Potassium, Na:Sodium, Zn:Zinc. The remainder are vitamins.

Output	Leafy Greens			Roots			Vegetables			Legumes			Cereals		
	RMSE	R^2^	PCC	RMSE	R^2^	PCC	RMSE	R^2^	PCC	RMSE	R^2^	PCC	RMSE	R^2^	PCC
Ca	23.97	0.82	0.90	7.54	0.90	0.95	73.06	0.41	0.98	21.88	0.55	0.88	7.57	0.50	0.92
Fe	0.29	0.82	0.90	0.24	0.56	0.75	0.15	0.76	0.86	0.69	0.49	0.71	0.39	0.52	0.77
Mg	8.95	0.80	0.89	4.65	0.83	0.96	5.12	0.85	0.93	10.72	0.70	0.84	11.03	0.58	0.81
Ph	11.37	0.66	0.83	10.33	0.82	0.89	24.90	0.44	0.58	26.92	0.65	0.85	27.41	0.48	0.79
K	76.50	0.70	0.84	71.64	0.88	0.94	68.81	−.12	0.72	73.35	0.49	0.79	58.98	−0.19	0.27
Na	23.85	0.68	0.80	9.90	0.81	0.91	4.92	0.50	0.98	9.02	0.11	0.88	19.06	0.78	0.92
Zn	0.10	0.72	0.86	0.09	−0.23	0.59	0.08	0.96	0.97	0.32	0.48	0.70	0.23	0.68	0.83
A	36.24	0.93	0.97	19.73	0.65	0.96	21.48	0.91	0.98	11.66	0.45	0.76	NA	NA	NA
C	11.97	0.65	0.84	6.65	0.85	0.94	7.05	0.88	0.96	3.71	0.77	0.94	NA	NA	NA
B1	0.02	0.84	0.92	0.01	0.92	0.99	0.01	0.85	0.92	0.05	0.64	0.80	0.04	0.66	0.85
B2	0.06	0.64	0.81	0.04	0.28	0.67	0.03	0.76	0.88	0.02	0.65	0.85	0.04	0.61	0.79
B3	0.16	0.76	0.93	0.12	0.82	0.92	0.27	0.69	0.82	0.19	0.64	0.85	0.49	0.69	0.85
B6	0.06	0.90	0.95	0.03	0.79	0.95	0.04	0.63	0.86	0.04	0.56	0.70	0.03	0.54	0.79
B9	20.90	0.63	0.80	7.18	0.82	0.97	4.90	0.89	0.95	29.38	0.68	0.83	19.88	0.43	0.91

Various metrics (R^2^,RMSE,PCC) by category for animal-based foods												
Output	Beef			Lamb			Chicken			Veal		
	RMSE	R^2^	PCC	RMSE	R^2^	PCC	RMSE	R^2^	PCC	RMSE	R^2^	PCC
Ca	1.85	0.76	0.88	1.93	0.87	0.83	2.27	0.04	0.59	3.52	0.68	0.78
Fe	0.13	0.89	0.95	0.16	0.25	0.67	0.08	0.84	0.95	0.22	−1.63	0.34
Mg	2.68	0.03	0.48	1.50	0.31	0.57	2.22	0.43	0.67	4.48	0.10	0.37
Ph	18.31	0.32	0.70	19.91	−0.44	0.48	25.51	−0.06	0.73	46.28	−1.86	−0.12
K	33.23	0.19	0.60	32.69	0.31	0.63	32.81	0.15	0.88	37.47	0.25	0.91
Na	9.50	0.25	0.65	7.15	−0.27	0.35	13.65	−0.51	0.46	14.97	−1.00	0.33
Zn	0.38	0.95	0.98	0.24	0.91	0.96	0.15	0.80	1.00	0.17	0.93	0.99
A	1.24	0.39	0.90	1.67	0.80	0.91	3.67	0.73	0.86			
B1	0.01	0.60	0.80	0.01	0.73	0.87	0.01	0.62	0.92	0.02	−0.29	0.56
B2	0.02	0.95	0.98	0.01	0.94	0.97	0.03	−0.10	0.94	0.06	−0.06	0.47
B3	0.47	0.79	0.89	0.40	0.70	0.84	0.85	0.82	0.95	0.41	0.92	0.97
B6	0.05	0.78	0.88	0.04	0.89	0.95	0.02	0.83	0.95	0.15	−1.24	0.47
B9	0.99	−0.63	0.54	1.74	0.67	0.83	0.91	−2.81	−0.64	2.62	−2.82	0.91

## 3 Results

### 3.1 Approximately 10% of SR legacy foods can be paired to be used in model training

The single ingredient foods that are either raw or cooked were found in 35% of the SR legacy data, and 30% of these were paired into raw and cooked samples. The final selection of 840 foods (or 425 pairs) is 10% of SR legacy data (Fig. 2A), with an unequal distribution of data pairs by food category (Fig. 2B). We identified an anomaly where the content of a micronutrient was more in the cooked food than in the raw food in 50% of the pairs on average across the 14 micronutrients. The anomaly was more severe for the animal-based foods (77% vs 23% pairs, respectively; see Supplementary materials). This was partially caused by the bias introduced by the data representation convention. For the animal-based foods, the non-anomalous pairs are 30% of the total pairs for unscaled data and increase to 70% for PINS-cholesterol scaled data, p-value<0.01. This is reasonable, since the anomaly is due to a concentration bias (nutrient content in cooked food is more than in raw food), which is mitigated by scaling. For the plant-based foods, there is no significant change (p-value>0.05) in non-anomalous pairs using the scaling methods for plant-based foods, since the issue is a dilution bias which is mitigated however this does not cause an anomaly (nutrient content in cooked food is more than in raw food). The comparison of non-anomalous pairs for animal and plant-based foods is shown in Fig. 2. The **Discussion** section explains the reasons for this differing effectiveness of the scaling methods in reducing the bias and suggests other possible causes for the bias.

### 3.2 Scaling improves model performance

We trained predictive models on variants of the datasets as explained in Methods. The dry heat models (broiling, grilling, roasting processes; 247 animal-based foods) and wet heat models (steaming, boiling; 178 plant-based foods) were trained on the unscaled data, which is not filtered for the anomalous condition, and on data from both the scaling methods which is filtered for non-anomalous data. We use the metric RMSE to compare model performance and confirm the hypotheses described in Methods. For the dry heat models, the average RMSE (for 13 predictions) was 20% lower when the model was trained on data scaled by the PINS-cholesterol method than data scaled using SCS method, which had 15% lower RMSE compared to the model trained on unscaled data. (As mentioned in the Methods, the model prediction results were not significantly different for the data scaled for a constant cholesterol content and scaled for a 5% loss. So the results are reported for the former). Although the model performance based on PINS data for iron and zinc has lower average RMSE than cholesterol, we consider the model trained on PINS-cholesterol as the best model since there is a mechanistic explanation described in **Methods**. For the wet heat models, the average RMSE was 35% lower when the model was trained on SCS data than that on unscaled data. These comparisons are shown in Table 1, and all results are in Supplementary materials and further analysis is in **Discussion**. The best model for the wet heat process is trained on SCS data and for the dry heat process it is trained on PINS-cholesterol data. We now compare results from the best predictive ML models to the baseline model.

### 3.3 The predictive model performs 43% and 18% better than using the standard USDA retention factor model for wet and dry heat processes, respectively

**Fig. 3:**
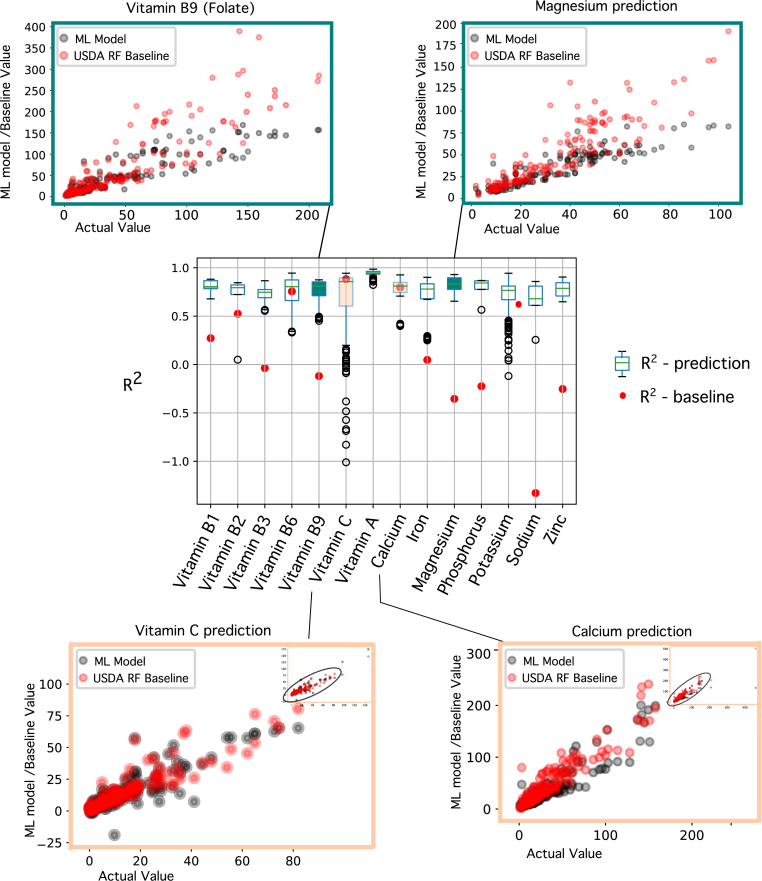
**Model Performance Analysis.** Centre: Comparing box plots of R^2^ (coefficient of determination) for the ML prediction models and R^2^ for the corresponding USDA baseline model. Details of the predicted values are shown in scatter plots, where the values from the prediction models and USDA baseline model are plotted against actual values (ground truth). In the top two scatter plots, the ML model performance is better than the baseline. Plots for vitamin B9 (folate) and magnesium show that the baseline model tends to have erroneously higher values than the predicted values, relative to the actual data. The lower two scatter plots are for the case where ML prediction was worse than baseline but only by a small margin. Plots for vitamin C and calcium have a noticeable overlap in values for the prediction model and baseline.

We compared the predicted concentrations of the micronutrients in the cooked foods for both the wet heat processes and the dry heat processes against the two baseline models, as described in the **Methods** section. When compared to the naïve baseline (i.e., retention factor is always 100%), the predictive model is better in all 27 out of the 27 comparisons (100%; RMSE of 9.90 ± 16.45 vs 31.29 ± 56.56, respectively; 64% decrease of RMSE on average for wet heat, p-value <0.01; 52% decrease in RMSE on average for dry heat, p-value <0.01). Then, to compare with the standard practice, we computed micronutrient concentrations using the USDA's Retention Factor table (see **Methods**) as shown in Table 2. In that case, the predictive model was better than this baseline in 22 out of the 27 comparisons (81%; RMSE of 9.90 ± 16.45 vs 16.45 ± 28.41, respectively; 43% decrease of RMSE on average for wet heat, p-value <0.01; 18% decrease in RMSE on average for dry heat, p-value <0.01). Fig. 3 depicts the correlation between predicted and actual (ground truth) values for the 14 micronutrients, for both the ML model and the USDA retention factor baseline. Next, we investigated the difference in the predictive performance when curating retention factors from literature. For this, we identified the retention factors of vitamin C (ascorbic acid) for 12 sample foods (green beans, beet greens, broccoli, Chinese cabbage, carrots, cauliflower, mustard greens, green peas, green peppers, pumpkin, spinach, zucchini) and of vitamin B9 (folate) for 12 sample foods (amaranth leaves, broccoli, drumstick leaves, snap beans, lentils, okra, onions, potatoes, green peas, soybeans spinach, taro leaves) (see Supplementary materials). In both cases, the ML model had a better agreement with the ground truth data than the Literature Retention factor baseline, although less so for vitamin C (for vitamin C (ascorbic acid), RMSE 10.51 vs 13.31, p-value = 0.026; for vitamin B9 (folate) RMSE 25.84 vs 97.22, p-value = 0.013). Note that retention factor information for each micronutrient is not available for the majority of foods, and it is a time consuming and expensive process to measure it. Using scale-invariant metrics reach the same conclusions (see Supplementary materials). The **Discussion** section elaborates further on the reasons that any RF baseline method is error prone and not appropriate to compute nutritional baselines.

### 3.4 Prediction performance is best for legumes, and worst for cereals, in the plant-based food categories, and best for beef and worst for veal in the animal-based food categories

**Fig. 4:**
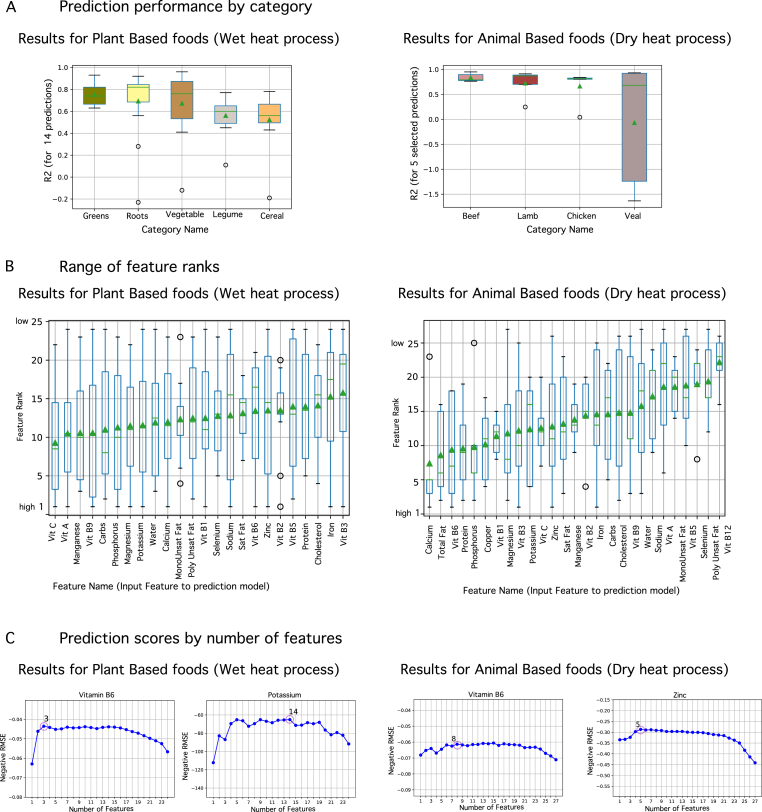
**Results.** (A): Box plot of R^2^ for predictions by food category. For the plant-based foods the box plot shows all 14 predictions. Leafy green vegetables have the best performance and Cereals the worst. For the animal-based foods, only five predictions are considered since they have the most reliable data as mentioned in Results. Beef has the best performance and veal is the worst (B): Box plot of feature ranks for the input features, where rank one is highest. Features are arranged in ascending order of average rank. Average ranks for both plant-based foods (and WH process) and animal-based foods (and DH process) are in the mid-range. No feature has a consistent high rank cross all the predictions. (C): Plots of performance-vs number of features. Vitamin B6 and potassium are shown as examples for the WH process and vitamin B6 and zinc for the DH process. The best features for vitamin B6 (WH) are vitamin B6, zinc, vitamin C. Best Features for potassium (WH) are potassium, vitamin B9 (folate), water, magnesium, vitamin A, saturated fats, vitamin B5 (pantothenic acid), vitamin B1 (thiamine), iron, poly unsaturated fats, selenium, vitamin B3 (niacin), vitamin B6 and zinc. Best Features for vitamin B6 (DH) are vitamin B6, magnesium, calcium, vitamin B2 (riboflavin), calcium, total fats, vit C and carbohydrates. Best features for zinc are zinc, phosphorus, calcium, potassium and total protein. The combined interpretation of B and C suggests that feature selection results differ for every nutrient prediction. (For interpretation of the references to colour in this figure legend, the reader is referred to the Web version of this article.)

As reported in prior literature, the food structure/phenotype influences the chemical and physical changes that occur in food processes. Here we use the food category to represent this concept and show the differences in predictability. We group the 14 predicted micronutrient values by the food category and calculated the R^2^ (Table 3 and Fig. 4A). Legumes have the highest average R^2^ of 0.75 ± 0.10 and cereals have the least average R^2^ of 0.52 ± 0.25. In the dry-heat processed animal-based foods, beef had the highest average with R^2^ of 0.48 ± 0.46 and veal the least average R^2^ of - 0.50 ± 1.21. Due to the uncertainty associated with methods of data generation, USDA specifies the nutrients with most reliable data, these are vitamin B3 (niacin), vitamin B6, calcium, iron and zinc. The highest average R^2^ is now 0.85 ± 0.08 for beef and the lowest is −0.06 ± 1.26 for veal. As such, the nutrient loss is better predicted in leafy greens and beef given the current training data.

### 3.5 High variability on the top predictive features

There is a notable lack of feature importance order across the prediction models. Fig. 3B shows the feature ranks, where the features are ordered by their average rank across predictions. The average rank is in the mid-range for both the WH and DH process, suggesting that no feature has a consistent importance across all the predictions**.** Fig. 3**C** shows performance by feature-size plots for vitamin B6 and potassium (WH) and vitamin B6 and zinc (DH) and the feature names are listed in the caption. The common observation is that the top ranked feature is the micronutrient itself in the raw food, as expected, but all other input features are specific to every prediction. The complete coverage of best features and feature ranks is in the **Supplementary materials**.

## 4 Discussion

The prior sections addressed the methods to building predictive ML models for the micronutrient content in cooked foods and the discovery of a data scaling method to remedy the bias of unknown yield factors. The results proved that this novel method outperforms the baseline method, which is significant since it offers the potential to scale across diverse foods without compromising the accuracy. However, realizing this potential, requires larger datasets than currently available. Accordingly, this section delves into the observed limitations of the SR Legacy dataset and interpretation of the results, with the aim of providing guidance to the future efforts of building larger food composition datasets (Fukagawa et al., 2022; Ahmed et al., 2022; Hinojosa-Nogueira et al., 2021; Desiere et al., 2001) since the data generation process is time consuming and expensive.

Regarding the predictive performance, we elaborate on some reasons for the lower performance of the baseline methods. The scatter plots in Fig. 3 for vitamin B9 (folate) and magnesium show that the baseline method overestimates the composition, which implies that the baseline RF is much greater than the RF inherent in the true data. RF represents the rate of loss which is influenced by process-related factors like processing times, surface area of vegetable exposed to processing conditions. Ideally for a fair comparison, these factors should be known for the baseline and matched to the data at hand. This can easily be addressed by recording additional meta-data. However, the more challenging discrepancy was that the baseline is a simple linear method, while the prediction model is a much more complex multiparametric non-linear ML model. Inevitably more sophisticated methods will emerge whether machine learning, mechanistic or a hybrid, and a suitable state-of-the-art baseline method will be available for comparison.

The current dataset has been the primary food composition dataset in the US for several decades, however it has several gaps in the data structure and data sampling that are regarded as necessary for datasets in current times. We assess these limitations to inform methods in building future datasets; the selection of food samples, recording of structured metadata/provenance, checking for data quality, and determining the composition features. The provenance of the data was incomplete in at least two different aspects. The composition data was calculated for some foods, and there was no explanation for the calculation method and no mention of the reference food/data used in the calculation method. It is unclear whether the samples for the raw and cooked food were related. Additionally, ontologies or structured vocabularies are a valuable resource when creating a format or structure for the dataset. Regarding data quality, we have described the anomalous condition in the **Results**. This is an example of a basic data sanity check, and especially in the context of a prediction hypotheses. Predictive performance depends on both the sample size as well as the entropy of the dataset, and one can use the predictive performance of the model as a guide for the sampling size for gathering new experimental data. There was only a single representative instance for each food and factors like geography, method of agriculture etc. are known to significantly impact the composition. The congruence of food-source and cooking method (plant-based foods were cooked by wet heat methods and animal-foods are cooked by dry heat methods) makes it impossible to compare model performance by either variable independently. While animal-based foods are often cooked in dry heat conditions, plant-based foods are also cooked by these methods, so this omission is also relevant to dietary representation. From the perspective of data modelling, it is especially disappointing, since we discovered that prediction performance varies by category within a given source. Such results could increase our knowledge of nutrient loss and designing prevention strategies, as well as provide hypotheses for greater food sampling. Regarding the feature space per sample, we suggest including process parameters and features known to influence nutrient loss such as pH.

Finally, we address some details of the anomaly caused by the representation of the composition per 100 g of food and unknown yield factors. This issue was mitigated by data scaling methods, however our observation show that this is not a complete resolution and new standards for data representation are required. The results from applying the scaling methods on the composition data, has two unrelated interpretations; the effect on the size of non-anomalous food-pairs (Fig. 3) and the effect on model performance trained on this data (Supplementary materials). As seen in Fig. 3, there is no significant effect (p-value = 0.06) for the plant-based foods where the data representation causes a dilution bias, and the anomaly could instead be due to different food samples used for the raw and cooked analysis. Whereas there is a significant effect (p-value <0.01) on animal-based foods where the anomaly is due to a concentration bias. Regarding the prediction performance, a few additional components used in the PINS method had good results besides the hypotheses. For plant-based foods, the performance for SCS data was the best, followed by carbohydrate PINS data. For animal-based foods, the performance by PINS-proteins data was the better than for zinc, iron and cholesterol. However, the results for PINS-carbohydrate and PINS-protein are likely due to the methods used for generating this data. Another possible solution might be to use yield factors when available, but since processing conditions are not available for SR data, we could use it. This analysis presents several questions for future inquiry, though the most important might be to ascertain a process-invariant nutrient and under which conditions and the biochemical/mechanistic explanation. This information might help for data transformations of existing data, but new data representation standards need to be considered and applied to future data generation efforts.

## 5 Conclusion

In conclusion, ML models have the potential to complement experimental methods in predicting the effects of food processing. Realizing this potential will require substantially more data than currently available, and with more meta-data to describe the food samples and the process. Such high-quality data would increase the reliability of these models to the extent of designing strategies in process control for desired composition. Ultimately the above objectives require a robust infrastructure that includes standardized datasets, the toolset to mine and utilize this data and a feedback loop from the data analysis and modelling to guide future data generation.

## CRediT authorship contribution statement

**Tarini Naravane:** Formal analysis, Data curation, Methodology, Writing - original draft. **Ilias Tagkopoulos:** Supervision, Conceptualization, Funding acquisition, Methodology, Writing - original draft.

## Declaration of competing interest

The authors declare that they have no known competing financial interests or personal relationships that could have appeared to influence the work reported in this paper.

## Data Availability

Data is included in the Supplementary material
